# Diversify, produce or buy? An analysis of factors contributing to household dietary diversity among shrimp and non-shrimp farmers in coastal Bangladesh

**DOI:** 10.1007/s12571-021-01245-w

**Published:** 2022-01-28

**Authors:** Amelie Bernzen, Ellen Mangnus, Franziska Sohns

**Affiliations:** 1 Vechta Institute of Sustainability Transformation in Rural Areas (VISTRA), University of Vechta, Faculty II – Geography, Vechta, Germany; 2 Wageningen University, Wageningen, Netherlands; 3 University of Greenwich, Department of International Business and Economics, Greenwich, UK

**Keywords:** Food and nutrition security, Dietary diversity, Crop diversification, Farmer livelihoods, Aquaculture, Coastal Bangladesh

## Abstract

Until the Covid-19 pandemic, Bangladesh had reported consistent improvements regarding its food and nutrition security (FNS) status, and yet, the country still features poor FNS outcomes among parts of its population. In rural coastal regions of the Ganges–Brahmaputra-Meghna delta, farming households’ vulnerability is particularly exacerbated by a range of environmental hazards, increasing challenges for agriculture to contribute to higher FNS levels. In the context of existing literature on the trade-offs between subsistence agriculture and cash-earning livelihood activities, vis-à-vis food and nutrition security outcomes, this article assesses the relative contribution of crop diversification vis à vis other factors on the households’ Food Consumption Score (FCS) in specific livelihood contexts. We provide differentiated analyses between primarily export-oriented shrimp farming and non-shrimp farming households, so policy makers can better address FNS targets. Quantitative data from 1,188 sample households across the delta were analysed through descriptive and linear regression analyses. Results show that households cultivating shrimp have a significantly higher dietary diversity than households that do not. Among shrimp farmers, crop diversification has the relatively strongest significant positive effect on dietary diversity, suggesting part of the aquacultural crops are geared towards subsistence. By contrast, crop diversification seems to have a negative effect on dietary diversity among households that do not produce shrimp, especially when different agricultural crops are combined. Importantly, both for shrimp and non-shrimp farmers, crop diversification systems combining agriculture with aquaculture, and agroforestry seem to improve diverse diets among households. While by no means a panacea to solving FNS challenges among rural households, we suggest that promoting specific crop diversification systems could be a beneficial pathway to improved FNS outcomes.

## Introduction

Despite improvements in food and nutrition security (FNS)^1^ over the last few decades, the prevalence of undernutrition remains high in the Global South. Importantly, undernutrition is not merely the result of low food quantities or calories consumed. The quality of the diet including the diversity of food groups consumed, are also of great importance. Bangladesh is a good example for this development: While consistent success in FNS was reported up until the Covid-19 pandemic (FAO, 2020), due in part to increasing agricultural productivity since the 1980s, the country was still ranked 86th out of 119 qualifying countries in the 2018 Global Hunger Index (GHI, 2018). Improving the quality and diversity of the diet among the overall population remains a major concern, with staple foods such as cereals dominating the Bangladeshi diet (Osmani et al., 2016).

As reflected in the United Nations’ 2030 Agenda for Sustainable Development, agriculture has a central role to play in combatting undernutrition and improving dietary diversity. Increasingly, countries give political priority to improving the nutritional impact of agricultural investments (Rampa & van Seters, 2013). At the same time, the question of how agriculture can best contribute to food and nutrition security remains debated, and is of particular importance to vulnerable individuals within farming households.

A number of frameworks highlight the dynamic and multifaceted linkages between agriculture, health, and nutrition (Herforth & Harris, 2014; IFPRI, 2011; Jaenicke & Virchow, 2013; Kadiyala et al., 2014). A popular framework is the Agriculture to Nutrition pathways framework developed by Ruel and Alderman (2013). Six pathways are identified through which agricultural interventions can impact nutrition: (1) food access from own production; (2) income from the sale of commodities produced; (3) food prices from changes in supply and demand; (4) women's social status and empowerment through increased access to and control over resources; (5) women's time through participation in agriculture, which can be either positive or negative for their own nutrition and that of their children; and (6) women's health and nutrition through engagement in agriculture, which can also have either positive or negative impacts, depending on exposure to toxic agents and the balance between energy intake and expenditure. However, evidence on the impact of different pathways remains ambiguous.

In this overall context, the aim of this paper is to help understand in which ways crop diversification plays a role, as it has been explored as one adaptation strategy for both market-oriented and subsistence-oriented smallholder farming households to reduce their livelihood vulnerability and increase the resilience of farm system against the effects of climate change and other external stressors (e.g. Lin, 2011; McCord et al., 2015). We here understand crops as plant or animal products cultivated by farmers for sale on the market or for subsistence, and crop diversification as “a process that makes a simplified cropping system more diverse in time and space by adding additional crops” (Hufnagel et al., 2020: 14 p. 4). Crop diversification can be analysed at different scales, starting at the plot level and moving to field, landscape, and regional scales. Of these, the plot and field scales are most relevant to farmer households: these include practices such as cultivating diverse genetic crop varieties; cultivating different species in parallel as polycultures; double cropping or intercropping; integrated mixed cropping systems (i.e. aquaculture or livestock with crops); and/or crop rotation systems (Kremen et al., 2012; Lin, 2011).

The ways in which crop diversification on farm level can contribute to household FNS has been evaluated in a number of studies (Islam et al., 2018). Systematic literature reviews on how agriculture can contribute to improved farm household FNS conclude that agricultural development programmes which promote crop diversity, micronutrient-rich crops, dairy, or small animal rearing can improve the production and consumption of targeted commodities, and in some cases that this leads to dietary diversity at the household level (Berti et al., 2004; DFID, 2014; Fiorella et al., 2016; Leroy et al., 2009; Masset et al., 2012; Oyarzun, et al., 2013; Pandey et al., 2016; Webb & Kennedy, 2014; Webb-Girard et al., 2012). The question that arises is which role crop diversification plays within the overall livelihood context: is it aimed primarily at increasing the number of cash crops or geared toward own consumption? A study on Bangladesh by Sraboni et al. (2014) found that crop diversity leads to increased dietary diversity if households consume some of the food that they produce. In line with this, negative correlations were found between smallholder food security and cash crop production (Anderman et al., 2014). However, other studies find that nutrition security can also be achieved by improved farmer income, which is often a result of selling cash-crops (Pierre Louis et al., 2007; Kuma et al., 2018).

How and to what extent capture fisheries and aquaculture contribute to improving nutrition, food security, and economic growth in developing and emergent countries has been highlighted in a number of studies. Béné et al. (2015) systematically reviewed 202 articles published between 2003 and 2014 and found that while fish contributes undeniably to nutrition and food security, the links between fisheries/aquaculture and poverty alleviation are complex and still unclear. Furthermore, Kawarazuka and Béné (2010) identify three pathways that exist between small scale fisheries and aquaculture-based livelihoods, and nutritional security. The first pathway is the direct contribution of fish consumption towards household nutrition, as the fish are rich in protein, as well as nutrients such as vitamin A, calcium, iron and zinc. The second relates to cash income gained through the sale of fish and aquaculture products that helps households to access other foods and to improve their overall dietary intake. There is ample evidence that aquaculture constitutes—in view of the increasing global and urban demand—a valuable commodity. This is especially true for Bangladesh, as we will explain below. Finally, they observe that an improved economic status of women through their involvement in aquaculture and/or fisheries-related activities (processing and trading) also leads to improved household nutritional security. They remark that evidence however is mostly anecdotal and that more research is necessary. These pathways reflect three of the pathways of the more general (i.e. not commodity-specific) framework by Ruel and Alderman (2013) addressed above.

Overall, the relationship between crop diversity of farms and the quality of the diets of the households managing those farms is a focus which has not yet been well established (Fiorella et al., 2016), and most studies provide no regionally or production type-based differentiation of results (e.g. Ahmed & Waibel, 2019; Islam et al., 2018). This specific knowledge on (regional and livelihood) circumstances, however, is essential to developing policy measures which increase impact on FNS outcomes. There is, therefore, a need to identify further the circumstances under which crop diversity has a positive effect on household FNS. Furthermore, while a number of studies have addressed rural livelihoods and FNS in Bangladesh—especially in the coastal shrimp farming zones—the topic has rarely been subject to rigorous analyses using large quantitative datasets.

To address this research gap, based on a range of multivariate linear regression models analysing household survey data collected in the coastal regions of the Ganges–Brahmaputra-Meghna delta in Bangladesh, we examine and compare the contribution of households’ crop diversification to FNS outcomes (measured through the household food consumption score, FCS), relative to demographic, socio-economic, physical, social, environmental and other livelihood-related factors, such as food production and consumption patterns. We address spatial—and to a lesser extent, temporal—crop diversification as our data covers only one wet and one dry season. We examine two distinct livelihood contexts: farmer households producing shrimp and/or prawn and those who do not.^2^ Among these two farming systems, the crop diversification options in shrimp farming operations are restricted to the extent that salt-intolerant crops (most vegetables, pulses, etc.) are mostly not cultivable in these systems, which can lead to an overall reduction in the number of different crops grown. Furthermore, our analysis seeks to acknowledge that crop diversification can be geared towards market or subsistence, or both, thereby also aiming to add to the understanding of the trade-offs between subsistence farming and cash-earning farming activities, vis-à-vis food and nutrition security outcomes. Cultivating a primarily export-oriented commodity, shrimp farming is—on average—more market-oriented than agricultural crop farming.

The remainder of the paper is organized as follows: The second section presents a review of the insights on the aquaculture-FNS relationship available in relevant literature in Bangladesh. The third section introduces methodology, while the empirical results are presented and discussed in the fourth section. The final section draws conclusions and implications.

## Food security and Aquaculture in Bangladesh

Even though Bangladesh has made significant progress in poverty alleviation in general and food security in specific—the percentage of Bangladeshis living beneath the 1.90$-a-day poverty line declined from 44.2% in 1991 to 13.8% in 2016/17 (World Bank, 2018)—still 13% of the Bangladeshi population is undernourished by FAO’s definition: not having access to adequate amounts of safe, nutritious food to sustain a healthy and productive life (FAOstat, 2020, 3-year average 2017–19). In addition, the prevalence of acute and chronic malnutrition among children under five years of age remains alarming, at an estimated 30.8% in 2018 (FAOstat, 2020). An estimated 18% of the country’s adult women are also acutely malnourished.

The government of Bangladesh is strongly focused on enhancing the country’s food and nutrition security by means of becoming self-sufficient. Since the 1980s, Bangladesh has increased its food production—especially in cereals—to the extent that it can today be called overall food self-sufficient in terms of calorie availability (Osmani et al., 2016). It’s Seventh Five-Year Plan (GED, 2015) aims, among other things, to achieve an adequate and stable supply of safe and nutritious food for all, especially women and children (Compact, 2025, 2016). The objective of the National Agriculture Policy aims to increase production of all crops and its National Nutrition Policy (NNP, 2015) seeks to improve the nutritional status of Bangladeshis by ensuring the availability of adequate and safe food, as well as the diversification of diets. It supports breastfeeding promotion programmes, and nutrition-sensitive interventions, such as agricultural programmes to promote micronutrient-rich foods. Yet, over the past decade, concerns have increased in light of a significant slowdown in agricultural growth rates (Osmani et al., 2016). Furthermore, it is estimated that “domestic production is increasingly unable to meet consumer demand for a more diversified diet, with a particular shortfall in the production of pulses and oilseeds” (JPGSPH, 2016: 48).

The fishery sector (including aquaculture, i.e. fish, shrimp, prawn, crabs and other crustaceans) is key in combatting food insecurity in Bangladesh. After rice, in value terms of share of the food budget, fish products are Bangladesh’s most important food (Reardon et al., 2014). They supplement about 60% of Bangladeshi people’s daily animal protein intake. Over the past decades, annual per capita domestic consumption of fish has increased from 13.4 kg in 2000 to 19.71 kg in 2016 (WorldFish, 2017). However, when disaggregating between locations it becomes clear that for urban households a stronger increase holds. In 2000, urban households consumed 10% more fish than rural households, in 2010 this was 31%. Nevertheless, overall, the poorest households increased their consumption by more than 57% (Rashid et al., 2016). Toufique and Belton (2014) relate this result to the increase in aquaculture production, which constituted more than half (57%) of total fish production in 2018–19 compared to 16% in 1983–84 (DoF, 2019).

The fisheries sector plays a very important role for the Bangladeshi economy, contributing 3.69% to the country’s Gross Domestic Product (GDP) and 22.60% to the agricultural GDP (FRSS, 2016). It is the second largest export industry after garments and textiles in terms of export value and produces 2.5% of the global production of shrimp (Shamsuzzaman et al., 2017). More than 17 million people including about 1.4 million women depend on the fisheries sector for their livelihoods through fishing, farming, fish handling, and processing (BFTI, 2016).

Shrimp and prawn are Bangladesh exports’ pride. In 1980, approximately 20,000 ha of land were being used for shrimp and prawn farming (Metcalfe, 2003). By 2016, this was 275,509 ha, meaning an increase of 1278% in area in only 30 years (FRSS, 2016). This impressive growth can be explained by the expanding global demand (Afroz & Alam, 2013; Swapan & Gavin, 2011), particularly of the EU, North America and Japan, in combination with a growing middle class in Bangladesh itself (Belton & Azad, 2012). Government support such as fiscal incentives for exports, preferential loan rates, tax holidays, income tax rebates and donor programmes also aided the expansion (e.g., Swapan & Gavin, 2011). Of the total fisheries export value, shrimp share ranged from 66-86% over the period 2007/08-2013/14 (DoF, 2015). Today many regions in the southwest, but especially the districts of Khulna, Bagerhat and Satkhira, are almost exclusively dedicated to shrimp farming, constituting up to 70% of the total area in some villages (Afroz & Alam, 2013: DoF, 2015).

The integration of Bangladeshi shrimp farming into international value chains entails not only economic advantages for the national economy and the coastal region, but also some very serious social and ecological problems such as decreasing biodiversity, water pollution, shortage of drinking water and in particular soil salinization are accumulating (Paul & Vogl, 2011; Sohel & Ullah, 2012). The latter makes agricultural land use practically impossible, as crops cannot tolerate the salt levels, which are often too high even for more salt-tolerant varieties. Shrimp cultivation also affects neighbouring lands as saline water seeps into the soil (Belton & Bush, 2014; Rahman et al., 2013; Paul & Vogl, 2013). In another study, Belton (2016) finds that self-sufficiency is undermined in the shrimp farming village of Salabunia, making the households increasingly vulnerable to food price shocks. He finds that this increasing dependency on the market stimulates men to migrate and engage in agricultural labour in exchange for rice. As a consequence, women are increasingly responsible for managing the shrimp ghers.

These developments are linked to a shift concerning the source of the fish consumed. Farmed fish consumption is increasingly purchased as opposed to home produced (data extracted from BBS, 2011). This implies that “commercial aquaculture” has moved to be far more important than subsistence fish farming (Hernandez et al., 2018). Studies report exclusion of poorer households which previously captured fishes from these areas during the monsoon (Toufique & Gregory, 2008). Toufique and Belton (2014) remark that previously caught SIS small fish are particularly rich sources of micronutrients including iron, zinc, calcium and vitamin A. These fishes have been threatened by the expansion of shrimp farming. Other farmed species are often bigger and too expensive for poor consumers. On the other hand, Ahmed and Waibel (2019) find that homestead aquaculture in Bangladesh increased household food consumption and improved dietary diversity by both increasing higher fish consumption and generating additional cash income. The additional income from aquaculture also contributed to an improved diet among small farmers as it was spent on protein rich and energy-dense food items. Belton et al. (2014) found that commercially oriented smallholder aquaculture producers consumed larger quantities of fish from their own farms than households operating subsistence-oriented fish production systems. On average, individuals from households practicing aquaculture consumed (and produced) more rice, fruits, non-leafy vegetables and fish per capita than those that did not. E-Jahan et al. (2010) suggest that aquaculture might make a greater contribution towards reducing the effects of poverty if the production of small fish was promoted along with carps.

Few studies explicitly address the relationship between crop diversity and food security outcomes in Bangladesh. In their panel study using nationally representative data, Islam et.al. (2018) find that besides farm diversification, market access, commercialisation of farms, off-farm income and women’s empowerment have positive and significant effects on household dietary diversity. However, they provide no differentiated analysis based on major crop types dominating household livelihoods. Finally, (nutrition) education is highlighted as an important factor to increasing household food and nutrition security, as demonstrated in a study by Baliki et al. (2019) on the long-term effects of an integrated home garden intervention in Bangladesh. Other studies by Dey et al. (2013) and Murshed-E-Jahan and Pemsl (2011) found that farmer training in integrated aquaculture-agriculture had a significant positive impact on farmers’ technical efficiency, total factor productivity and net incomes, which resulted in higher food consumption and better household nutrition.

We now present the study site selection and methodology that we employ to provide a deeper understanding of how crop diversification (and other factors) can contribute to FNS in shrimp cultivation and non-shrimp farming livelihood contexts, respectively.

## Methodology

This paper presents findings from a secondary analysis of a dataset that was collected through the University of Cologne (UoC) in the context of the Belmont Forum project "BanD-AID" and kindly provided to the authors by the grant recipients. The first author of this paper, Amelie Bernzen, was formerly affiliated with the UoC and researcher within the BanD-AID consortium. She was involved in the design and collection of the empirical survey data analysed here.

### Study site selection and household survey

**Fig. 1:**
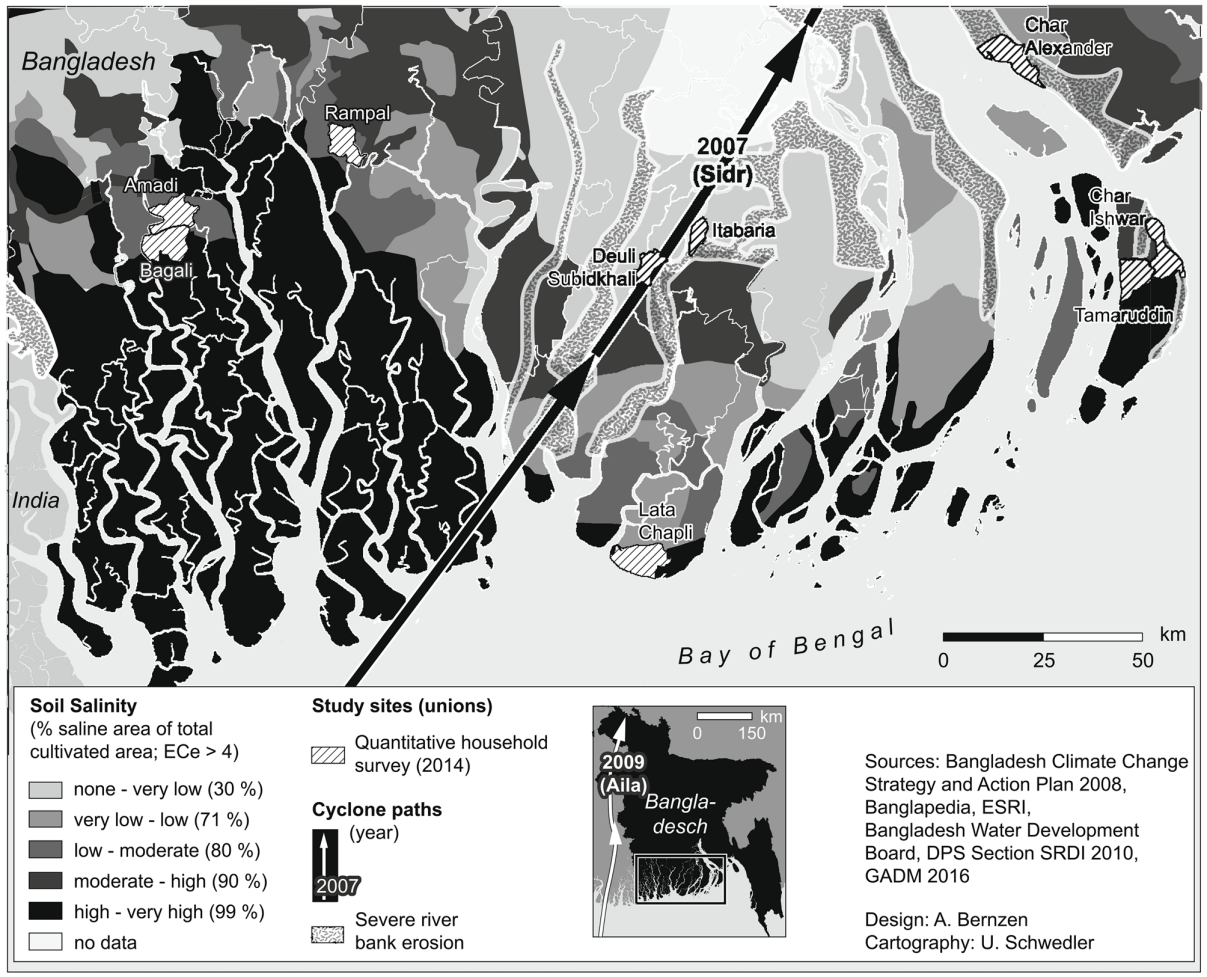
Soil salinity, Sidr and Aila cyclone paths, riverbank erosion risk areas and study sites in coastal Bangladesh (own design, slightly adapted from Bernzen et al., 2019)

Standardized household interviews with fixed and open-ended items were conducted in late 2014 in nine *union parishads* (in five districts; three divisions), each consisting of several settlements. Union parishads are the smallest governmental administrative unit in Bangladesh for which census data is available. The unions (located on the map in Fig. 1) were purposefully selected to represent rural communities on polders in different areas of the Ganges-Meghna-Brahmaputra delta. This delta constitutes one of the most vulnerable regions worldwide to environmental hazards, growing populations, high poverty levels and scarce resources, and features unique morphological dynamics that cannot be found along the eastern Chittagong coast of Bangladesh. Importantly, the unions included both those which are dominated by shrimp farming and those which feature predominantly agricultural crop-based systems (paddy, pulse, fruit, vegetables). The unions were further selected to represent a broad variety of geographic, socio-economic, coastal and environmental circumstances, covering four bio-ecological zones and thereby the diversity of the region (see Appendix (Table 3)). Finally, pragmatic and logistical reasons contributed to the selection of the unions, like accessibility and existing contacts.

**Fig. 2:**
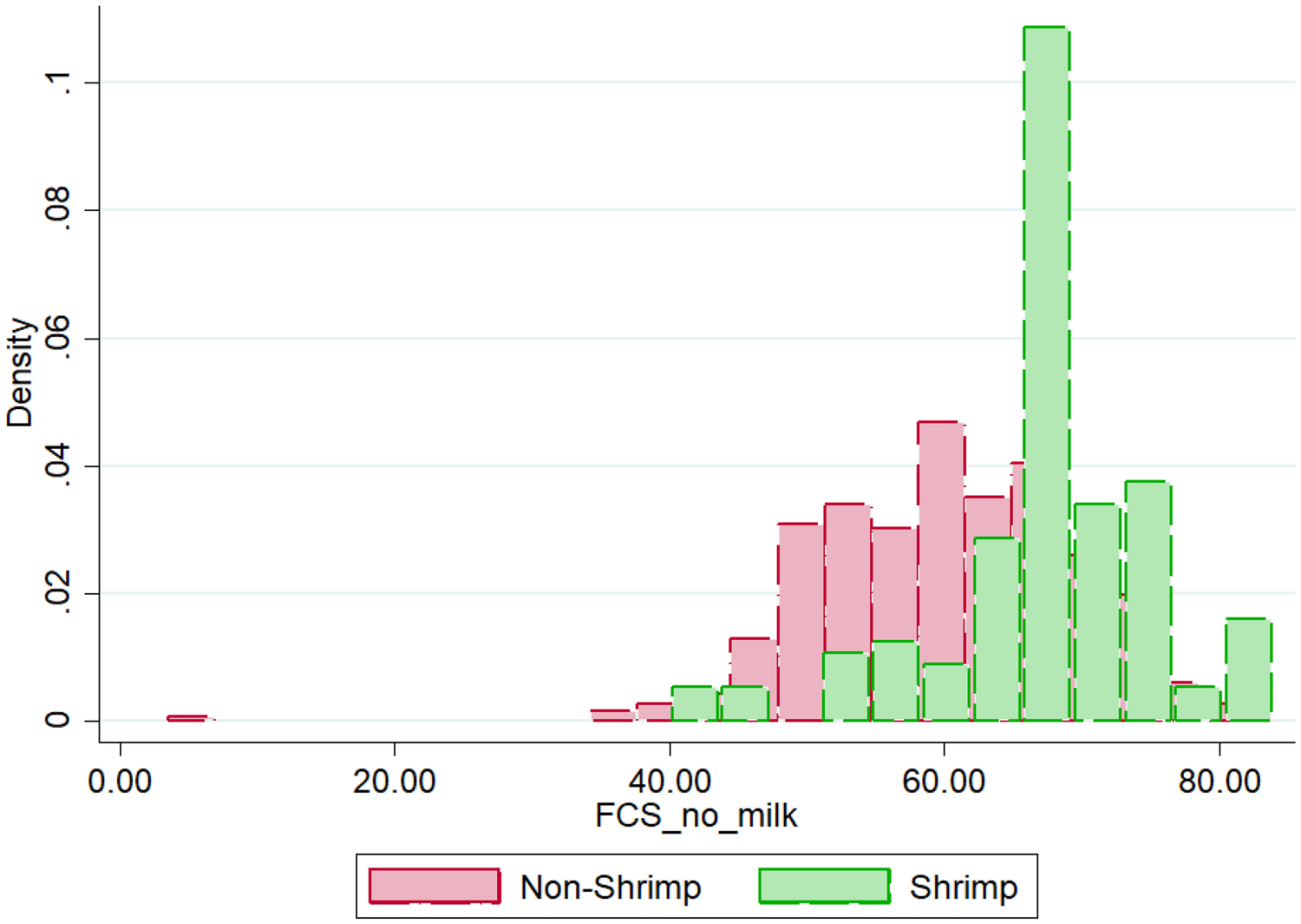
Distribution of Food Consumption Score by Production Decision

Within the unions, the target sample size for interviews in each study site was 150 households, generating subsamples which in themselves allow for relatively meaningful statistical analyses. This captured between 1.7% and 2.8% of the *union’*s population, respectively. Participating households were further selected on the basis of a minimum residence period of 10 years to capture possible land use changes and to reflect diversification. To (geographically) cover the different locations of the settled *union* area, trained local male and female field assistants were asked to identify all villages across the union, allocate a relatively similar target subsample (quota of the 150) to each and approach households by moving from the centre of the settlement (e.g. market square) towards the outer edges of the settlement along a major road/track—depending on the size of the village—interviewing every third to fifth household face-to-face. The final sample was 1,188 households. Interviews were held in most cases with the household head (85.3% of respondents: 973 males, 40 females) or his/her spouse (11.6% of respondents: 2 males, 136 female). Overall, however, while covering a large variety of rural livelihood settings across the delta, the sampling strategy does not generate a representative sample of all coastal households in Bangladesh.

### Empirical framework: Dependent and independent variables

The dependent variable in this study is diet quality, the extent to which nutritional needs are being met. One simple, commonly used measure that serves as a proxy for diet quality, or the extent to which nutritional needs are being met, is dietary diversity, which is associated with a range of benefits including greater and more adequate energy and nutrient intakes (e.g. Steyn et al., 2006). Average household diets in low-income countries are often limited to one or two starchy staple foods and may be especially lacking in micronutrient-rich fruits, vegetables and animal-source foods.

One indicator of dietary diversity on household level, which has been widely applied, is the food consumption score (FCS), developed by the World Food Programme (Carletto et al., 2013). This is "calculated using the frequency of consumption of different food groups consumed by a household/individual during the 7 days before the survey" (WFP VAM, 2008).^3^ As it captures the frequency of food group intake, it is seen as more adequate compared to other indicators which rely merely on food group count (Wiesmann et al., 2009). We constructed a modified FCS to determine relative differences in household dietary diversity across the study sites and household types (Carletto et al., 2013). The dataset provided information on surveyed households regarding the seven-day frequency of consumption in an average week for nine different food items which represent seven of the nine food groups typically included in FCS calculations. Rice, wheat, other cereals were included as "main staples"; meat, fish and seafood were included in the food group "meat and fish"; furthermore, we had data for the food groups "pulses," "fruit," "vegetables" and “sugar” (WFP VAM, 2008). Frequencies were measured as never (0), hardly at all/one day per week (1), every second day (3), most days (5) and daily (7). The survey did not include data for dairy/milk and oil. Oil is assumed to be consumed by all households in the sample on a daily basis, given the high importance of oil in the Bangladeshi diet (see FSC, 2009, Belton et al., 2014), and we therefore re-coded each household with "daily (7)" consumption. Consumption of milk varies somewhat depending on the income quintile; but is not an important source of protein, particularly for low-income households (Belton et al., 2014, FSC, 2009). This is also because milk is not commonly available in many parts of Bangladesh, leading it to be excluded in some calculations of dietary diversity scores in the country (e.g. in the study by Thorne-Lyman et al., 2010). Protein intake is therefore captured only through the food groups main staples, pulses and fish/meat. Given the high importance of rice in the Bangladeshi diet, it constitutes the major source of protein, even though fish, meat and dairy have a much higher protein content. Meat and fish are also significant sources of micronutrients (FAO, 2020).

Given these caveats, we use the following four food consumption groups, which are adapted to average Bangladeshi diets (FSC, 2009), as broader frames of reference: Poor consumption (FSC of 28 and lower), Borderline Consumption (> 28 and below 42), Acceptable low (42–52), Acceptable high (> 52).

Our independent variables from the dataset (see Table 1) were selected based on the literature review and include general demographics, such as the age of the household head measured in years (mean: 51 years), and the household head’s education. The latter was operationalized as a dichotomous variable with a value of 1 indicating household heads who at least obtained secondary education (19%). In addition, the independent variables cover socio-economic factors such as the monthly household income per capita measured in 100 taka (mean: 33.9), as well as off-farm income activities. The latter was operationalized as a dichotomous variable with a value of 1 indicating households that rely on off-farm activities as primary income source (41%). Moreover, the independent variables account for physical assets, such as owned and leased land measured in acres (mean: 3.22). Besides, the independent variables include agricultural strategies, such as the use of crop diversification and previous land use changes. Both variables were operationalized as a dichotomous variable with a value of 1 indicating, respectively, households that apply some kind of crop diversification (33%), and have changed the use of their land between 2000 and 2015 (26%). In addition, the independent variables cover exposure to potential environmental risks, such as the distance to the closest larger river or coast measured in km (mean: 1.24 km), arable land loss (mainly due to erosion), and salinization. The latter two variables were operationalized as a dichotomous variable with a value of 1 indicating, respectively, households that lost arable land in the past 10 years (19%), and households that indicate salinization as an issue in their village (27%). Moreover, the independent variables account for food production and consumption patterns, such the share of food produced on-farm that is consumed by the household (subsistence food consumption measured in %; mean: 63%), market dependency for food, i.e. the share of all consumed food by the household that is purchased on the market (commercial food consumption measured in %; mean: 51%), the decision-making power of the household head’s wife, which was operationalized as a dichotomous variable with a value of 1 indicating households in which the wife is part of the decision-making process for land use (7%), and non-shrimp farming, also operationalized as a dichotomous variable with a value of 1 indicating households that do not participate in shrimp farming (78%). Finally, the independent variables include social capital, such as the time period a household has lived in the village measured in years (mean: 44 years), the helpfulness of NGOs, the government, and neighbours. The latter three variables were operationalized as a dichotomous variable with a value of 1 indicating households that believe that these actors were of greatest help during the last natural disaster (NGO: 46%, government: 63%, neighbours: 27%).

**Table 1:** Descriptive statistics of independent variables

Variable	Mean	Std. Dev	Min	Max	Justification (e.g.)
General Demographics					
Age of HH (in years)	51.07	13.25	19	110	Sraboni et al., 2014
Education of HH (secondary and higher = 1)	0.19	0.40	0	1	Sraboni et al., 2014
Socio-economic Factors					
Primary income activity (off-farm = 1)	0.41	0.49	0	1	Belton et al., 2014
Household income per capita per month (in 100 taka)	33.90	29.44	3.75	400	Ruel & Alderman, 2013; Kuma, 2018
Physical Assets					
Accessible land (in acre)	3.22	7.13	0.03	88.92	Belton et al., 2014
Agricultural Strategies					
Crop diversification (yes = 1)	0.33	0.47	0	1	Sibhatu et al., 2015
Land use changes between 2000 and 2015 (yes = 1)	0.26	0.44	0	1	Toufique & Belton, 2014
Food Production and Consumption					
Share of subsistence food consumption (in %)	62.52	24.93	0	100	Ruel & Alderman, 2013
Share of commercial food consumption (in %)	51.42	23.34	0	100	Ruel & Alderman, 2013
Wife involved in land use decision making (yes = 1)	0.07	0.26	0	1	Harris-Fry et al., 2015, Sraboni et al., 2014
Non-shrimp farming (yes = 1)	0.78	0.41	0	1	Belton et al., 2014
Environmental Risks					
Arable land lost (yes = 1)	0.19	0.39	0	1	JPGSPH, 2016
Distance to closest river or coast (in km)	1.24	1.14	0.01	4.54	JPGSPH, 2016; Belton et al., 2014
Salinization (yes = 1)	0.27	0.44	0	1	Belton et al., 2014
Social Capital					
Time household has lived in village (in years)	44.22	17.62	0	100	Ali, 2005
Neighbours most helpful in crisis (yes = 1)	0.27	0.44	0	1	Ali, 2005
NGOs most helpful in crisis (yes = 1)	0.46	0.50	0	1	Ali, 2005
Government most helpful in crisis (yes = 1)	0.63	0.48	0	1	JPGSPH, 2016

*n* = 705

**Fig. 3:**
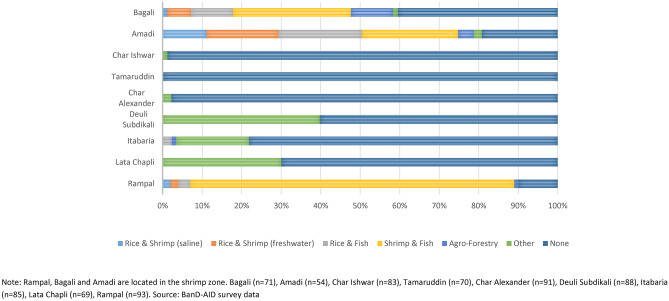
Share of households per union applying different types of crop diversification in 2014

To better understand and interpret the variable "crop diversification," it is helpful to understand which different crop diversification options were recorded in our study sites: Rice & Shrimp (bagda), Rice & Prawn (golda), Rice & Fish, Shrimp (bagda) & Fish, Agro-Forestry, and Other (Appendix (Fig. 3); multiple response possible). They therefore encompass different types of crop diversification measures (temporal and spatial), including crop rotation, double/multiple cropping, intercropping, variety mixtures and mixed cropping (for a detailed overview of different measures identified in the literature, see Hufnagel et al., 2020). For the south western study sites, they capture the common practices of shrimp (bagda) and prawn (golda) cultivation options which have been extensively discussed elsewhere (e.g. Ahmed, 2013; Belton, 2016). Essentially, depending on the local salinity levels, three variations of shrimp farming are possible (shrimp/rice alternately, shrimp/salt alternately, and shrimp only in cases where salinity levels are too high to farm rice in the wet season). This is different from prawn cultivation in combination with rice fields, which is either integrated (concurrent) or alternate (rotational). Fish are mostly farmed alongside prawn and shrimp in the same *gher *(field enclosed by embankments). A mixed cropping system of shrimp and prawn (e.g. Ahmed, 2013) was not observable on our study sites. “Other” crop diversification options include primarily intercropping of various agricultural crops. In more recent years, the agricultural crop farming regions east of the predominantly shrimp-farming districts have seen an increase in seasonally alternative crops: watermelons or sunflower seeds have been observed in Patuakhali district, for example. Agroforestry (growing trees and other plant or animal crops on the same plot, including aquasilviculture) has a long tradition in Bangladesh, providing homesteads with a range of crops, wood, fodder for livestock, and building materials. Further benefits of agroforestry include increased biodiversity and a slowed salinization process (Rahman et al. 2011).

That crop diversification options in shrimp farming operations are restricted because salt-intolerant crops (most vegetables, pulses, oil seeds etc.) are mostly not cultivable in these systems, leading to an overall reduction in the number of different crops grown, is also reflected in the data. Appendix (Table 4) shows that, while shrimp farmers sell more crops on average (2.32) than non-shrimp farmers (1.67), the overall variety of different crops sold on the market is much higher among non-shrimp farmers.

### Statistical modelling of Food Consumption Score (FCS)

To investigate the relative impact of the selected independent variables on households’ food and nutrition outcomes (here: FCS), we developed a range of multivariate linear regression models, using households as subjects of observation. Moreover, we calculated interaction effects between the variable “non-shrimp farming” (i.e. cultivates neither shrimp nor prawn) and the other independent variables. Using interaction terms is a widely used method to compare the effects of independent variables on a dependent variable between two groups (Karaca-Mandic et al., 2012). Hence, they help to understand how the effect of the independent variables on the food consumption score varies based on households’ production decisions (i.e. shrimp or no shrimp). We believe this to be necessary due to significant differences in the descriptive statistics among households that engage in shrimp farming and households that do not engage in shrimp farming (see Appendix (Table 5)). As we are interested in the role of agriculture on FNS, we looked only at households that hold and/or cultivate land and do not show missing values, leaving a total of 705 cases for the analysis.

In total, we fitted three linear regression models generated by following a stepwise procedure, introducing variables in succession (Heinze et al., 2018). In the first step, we added the variable “non-shrimp farming” in order to estimate the statistical difference in the FCS between households that engage in shrimp farming and households that do not engage in shrimp farming (m1). In a second step, we added the remaining independent variables (m2). In a third step, we inserted the interaction effect capturing the association between the independent variables and shrimp/non-shrimp farming (m3). The resulting multivariate linear regression model is represented in the following equation:

$${y}_{i}={\beta }_{0}+{\beta }_{p}{X}_{pi}+{\beta }_{q}{X}_{qi}+{\beta }_{q}{X}_{qi}*{\beta }_{p}{X}_{pi}+{\varepsilon }_{i}$$

with yi representing the estimated FCS of observation i. In addition, ${\beta }_{0}$ represents the constant term of the regression. Furthermore, ${\beta }_{p}$ represents the coefficient of the variable “shrimp/non-shrimp farming” (${X}_{pi}$), while ${\beta }_{q}$ represents the coefficients of the other independent variables (${X}_{qi}$). Moreover, the term ${\beta }_{q}{X}_{qi}*{\beta }_{p}{X}_{pi}$ represents the interaction effects between the variable “shrimp/non-shrimp farming” and the other independent variables, indicating whether the effects of the independent variables differ between households that engage in shrimp farming and those households that do not. Finally, ${\varepsilon }_{i}$ represents the error term of the equation.

We estimated robust standard errors to account for heteroscedasticity in the model’s unexplained variation and potential outliers (Pollet & Van Der Meij, 2017). In addition, we estimated both unstandardized regression coefficients as well as the standardised regression coefficients. While unstandardized regression coefficients represent the amount by which the dependent variable changes as a result of changes in an independent variable by one unit, standardised regression coefficients allow to rank independent variables based on their relative importance. Moreover, we checked for multicollinearity between the independent variables, which, with an VIF of 1.5, can be ruled out (Akinwande et al., 2015).

## Results and discussion

Overall, the data indicate that, with an average FCS of 62, the average household in this sample can be seen as having an “acceptable high” FCS. In line with this finding, 80% of the sampled households exhibit an “acceptable” FCS, indicating a right-skewed distribution. In contrast, 18% of the households have an “acceptable low” FCS, while only 2% of the households show a “poor/borderline” FCS. These figures are broadly comparable with official statistics on division level published in a 2015 report on the State of Food Security and Nutrition in Bangladesh (JPGSPH, 2016: Chittagong division: poor/borderline: 3% / acceptable low: 10% / Acceptable high: 87%; Barisal: 8% / 18% / 74%; Khulna: 14% / 13% / 73%). Interestingly, the data indicates a difference in the average FCS between households that engage in shrimp farming and households that do not engage in shrimp farming. While the former exhibit an average FCS of 67, the latter show an average FCS of 60. This difference can be seen as statistically significant (Mann–Whitney U-Test: 0.000) (Fig. 2).

The first regression model (m1, Table 2) confirms this descriptive finding. It indicates that households that do not engage in shrimp farming exhibit a significantly lower average FCS score (-7.3 points) in comparison to households that do engage in shrimp farming. Interestingly, the negative effect of “non-shrimp farming” on the FCS becomes insignificant after taking the other independent variables into account (m2). This indicates that some of the other independent variables might capture the effect. For instance, households that engage in shrimp farming show a higher monthly household income per capita, a higher share of commercial food consumption, and see salinization as an issue in their village more often (see Appendix (Table 5)). Therefore, it is possible that these variables drive the FCS, rather than shrimp-farming per se.

**Table 2:** Multivariate Linear Regression Results

Independent variables	m1	m1 (beta)	m2	m2 (beta)	m3	m3 (beta)
General Demographics						
Age of HH (in years)			0.043 (0.028)	0.061	0.062 (0.075)	0.088
Education of HH (secondary and higher = 1)			0.971 (0.815)	0.041	0.223 1.408	0.009
Socio-economic Factors						
Primary income activity (off-farm = 1)			-0.503 (0.627)	-0.027	1.668 (1.318)	0.088
Household income per capita per month (in 100 taka)			0.056*** *(0.012)*	0.177***	0.036 (0.023)	0.114
Physical Assets						
Accessible land (in acre)			0.128** *(0.051)*	0.097**	0.123** *(0.054)*	0.094**
Agricultural Strategies						
Crop diversification (yes = 1)			1.415 (0.956)	0.071	10.318*** *(2.514)*	0.519***
Land use changes between 2000 and 2015 (yes = 1)			-0.863 (0.817)	-0.040	-1.258 (1.680)	-0.059
Food Production and Consumption						
Share of subsistence food consumption (in %)			0.036** *(0.015)*	0.096**	0.074** *(0.029)*	0.198**
Share of commercial food consumption (in %)			0.039** *(0.017)*	0.097**	-0.065 (0.039)	-0.162
Wife involved in land use decision making (yes = 1)			2.972** *(1.181)*	0.083**	2.094 (2.208)	0.058
Non-shrimp farming (yes = 1)	-7.315*** *(0.757)*	0.323***	-0.873 (1.279)	-0.039	-1.081 (5.677)	-0.048
Environmental Risks						
Arable land lost (yes = 1)			-1.207 (0.849)	-0.051	-0.688 (4.148)	-0.029
Distance to closest river or coast (in km)			-0.177 (0.338)	0.022	0.003 (0.449)	0.000
Salinization (yes = 1)			4.908*** *(0.781)*	0.233***	1.507 (1.343)	0.072
Social Capital						
Time household has lived in village (in years)			-0.001 (0.023)	-0.003	-0.030 (0.056)	-0.056
Neighbours most helpful in crisis (yes = 1)			0.680 (0.867)	0.032	0.953 (1.283)	0.045
NGOs most helpful in crisis (yes = 1)			-3.040*** *(0.796)*	-0.162***	-5.164** *(1.988)*	-0.276**
Government most helpful in crisis (yes = 1)			-0.174 (0.722)	-0.009	3.654*** *(1.346)*	0.189***
Interaction Effects						
Age * Non-shrimp					-0.020 (0.080)	-0.052
Education * Non-shrimp					0.519 (1.716)	0.0186
Primary income activity * Non-shrimp					-2.463* *(1.489)*	-0.126*
Household income * Non-shrimp					0.019 (0.027)	0.056
Accessible land * Non-shrimp					0.613*** *(0.185)*	0.135***
Crop diversification * Non-shrimp					-10.462*** *(2.694)*	-0.379***
Land use changes * Non-shrimp					0.253 (1.945)	0.009
Subsistence food consumption * Non-shrimp					-0.044 (0.033)	-0.161
Commercial food consumption * Non-shrimp					0.125*** *(0.044)*	0.376***
Wife involved * Non-shrimp					1.212 (2.607)	0.031
Arable land lost * Non-shrimp					-0.287 (4.243)	-0.012
Distance to closest river or coast * Non-shrimp					-0.351 (0.751)	-0.028
Salinization * Non-shrimp					4.051** *(1.664)*	0.144**
Time in village * Non-shrimp					0.026 (0.061)	0.0654
Neighbours most helpful in crisis * Non-shrimp					-0.951 (1.684)	-0.039
NGOs most helpful in crisis * Non-shrimp					3.252 (2.170)	0.172
Government most helpful in crisis * Non-shrimp					-4.159*** *(1.584)*	-0.222***
constant	67.369*** (0.652)		53.646*** (2.413)		52.413*** (5.066)	
prob > chi^2^	0.000		0.000		0.000	
R^2^	0.104		0.290		0.341	
observations	705		705		705	

The relative importance of the different effects on the FCS for all households are revealed by comparing the standardized regression coefficients (beta) of the second model (m2). While a positive beta indicates a positive effect of the independent variable on the FCS, a negative beta indicates a negative effect of the independent variable on the FCS. The standardized regression coefficients indicate that, overall, salinization can be seen as the most important significant factor (0.233) explaining the FCS, followed by the monthly household income (0.177), and help received from NGOs (-0.162). In contrast, accessible land (0.097), higher market dependency for food (commercial food consumption share) (0.097), higher shares of produced food for subsistence (subsistence food consumption) (0.096), and a wife being part of the decision-making process (0.083) seem to be less important, but, nevertheless, significant. These results in part are comparable to those by Sraboni et al. (2014), that find that for rural Bangladesh, overall household wealth, education, and occupation have a stronger effect on adults’ nutritional status than women’s empowerment. In contrast to Islam et al. (2018), however, these overall findings do not indicate crop diversification to be a superior strategy to income-generating activities.

However, we clearly see a variation in the importance and ranking of these effects when assessing them in a more differentiated manner for shrimp and non-shrimp farmers. A comparison of the standardized regression coefficients (beta) of the third model (m3) reveals that, with regards to shrimp-producing households, crop diversification can be seen as the most important factor (0.519) explaining the FCS, followed by help received from NGOs (-0.276), share of subsistence food production (0.198) and government aid (0.189). In contrast, accessible land (0.094) seems to be less important, but, nevertheless, significant. In addition, it also indicates that, with regards to the difference in the effect of the independent variables on the FCS between shrimp-producing and non-shrimp-producing households, crop diversification (-0.379) and commercial food consumption (0.376) can be seen as the most important differentiators, followed by help received from the government (-0.222), salinization (0.144), size of accessible land (0.135), and off-farm activities (-0.126). We now discuss these significant effects in greater detail, starting with the most important in the overall model (beta).

First, ‘m2’ shows a general significant positive effect of salinization on the FCS. Interestingly, ‘m3’ indicates that salinization has no significant effect on the FCS of shrimp-producing households, but a significantly stronger, and presumably positive, effect on households that do not engage in shrimp production. This may seem counterintuitive at first, given that yields of salt-intolerant but nutritious crops such as rice, vegetables and fruit are much lower in saline areas than in freshwater ones (Belton et al., 2014). For non-shrimp farmers in the shrimp-dominated zones, we could here see an effect of Mozambique tilapia availability as an easily caught, invasive species that is present in all the canals and shrimp ponds in the saline zone in huge numbers, but not in the freshwater zone. This makes them cheaper than any other type of fish, very affordable even to poorer households in the saline zones—and can also be easily caught in canals—thereby increasing the share of households in these areas who consume fish on a daily basis (Belton et al., 2014).

Secondly, ‘m2’ indicates a general significant positive effect of the monthly household income per capita on the FCS. Interestingly, the third regression model (m3) does not indicate a significant interaction effect. Therefore, it can be assumed that the positive effect of the monthly household income applies in a more general manner. This supports other studies (e.g. Harris-Fry et al., 2015; Kuma et al., 2018) that highlight that income increases access to appropriate quantities of quality foods, which can complement produce from a farmer’s own production. This could include most types of salt-intolerant crops among shrimp farming households. The data reveal that shrimp farming households in the saline zone have higher average incomes per person than households in the freshwater zone and may hence be able to offset (or more than offset) lower levels of subsistence production (Appendix (Table 5)) . Moreover, although ‘m2’ does not indicate a general significant effect of off-farm activities as the primary source of income on the FCS, ‘m3’ reveals a significant interaction effect. Interestingly, ‘m3’ indicates that off-farm activities have no significant effect on the FCS of shrimp-producing households, but a significantly stronger negative effect on households that do not engage in shrimp production. For non-shrimp farmers in the saline zones, this may show the effect of salinity undermining subsistence capacity. Belton et al. (2014) have shown that this can lead to a large number of female household members working off-farm under highly unfavourable conditions. Overall, about half (45%, see Appendix (Table 5)) of the non-shrimp-farming households in our sample stated that off-farm activities are their primary source of income. In contrast to studies which have pointed to the benefits of overall livelihood diversification strategies, including off-farm income, our findings suggest that a household’s primary reliance on off-farm income does not necessarily improve household FNS. Recently, some authors have identified a need for further research to unpack this relationship, especially outside the shrimp farming zones (Roy & Basu, 2020). Our findings point in the same direction as earlier work by Sohns and Revilla-Diez (2017) which shows that off-farm activities cannot be seen as a panacea to improve household’s livelihoods in agriculture-dominated areas of developing countries, taking the example of Vietnam.

Third, ‘m2’ indicates a general significant negative effect of seeing NGOs as most helpful during environmental crises on the FCS. Likewise, m3 suggests a significant negative effect of seeing NGOs as most helpful on the FCS of shrimp-producing households. In addition, it does not indicate a significantly interaction effect. Therefore, it can be concluded that the negative effect applies to both: households that produce shrimps and households that do not produce shrimps. Regarding government aid, although ‘m2’ does not indicate a general significant effect of seeing it as most helpful during environmental crises on the FCS, ‘m3’ reveals a significant positive effect of seeing the government as most helpful on the FCS of shrimp-producing households. Interestingly, this positive effect appears to be significantly weaker, presumably even negative, for households that do not engage in shrimp production. Households’ reliance on NGO aid in times of crisis as the third most important but negative effect on the FCS among all households may not point per se to these organizations’ failure to support families after major climatic or environmental disasters. For example, Paul (2013) remarks that the shrimp zones’ relative proximity to the large urban centre of Khulna is associated with a larger number of permanent NGO offices in the region, including field staff to provide training and better agricultural extension. Rather, our result could indirectly imply that it is the most vulnerable and poor families reliant on external help who are the least food secure. By contrast, the important positive effect of government aid, perhaps not limited to but particularly concentrated among shrimp farmers, could be related to the relatively higher presence and interest of the government to support the shrimp farming economy in the saline zones, given the importance of shrimp exports to the Bangladeshi economy (Swapan & Gavin, 2011). That said, greater access to government aid among shrimp farmers could also be indicative of their overall superior social and political capital, rather than government presence per se.

Moreover, ‘m2’ shows a general significant positive effect of the size of accessible land on the FCS. Interestingly, ‘m3’ indicates that, while this positive effect applies to shrimp-producing households, it is even stronger for households that do not engage in shrimp farming. This could be explained by the general structure of the respective shrimp / cropping systems. Shrimp ponds are already quite extensive in size and additional land has a lower relative effect on additional income generation (i.e. higher volume shrimp production with some additional fish) than for crop farmers, where additional land could enable opportunities like including a pond, more diversified (cash crop) production or a larger kitchen garden on the property.

Although ‘m2’ does not indicate a general significant effect of crop diversification on the FCS, ‘m3’ reveals that crop diversification has a significant positive effect on the FCS of shrimp-producing households, and the relatively most important one. Interestingly, this effect is significantly weaker, presumably even negative, for households that do not produce shrimps. In addition, ‘m2’ suggests a general significant positive effect of the share of subsistence food consumption, i.e. the degree to which on-farm crop production is directed to self-subsistence (rather than cash-crop) on the FCS. Likewise, ‘m3’ shows that the share of subsistence food consumption has a significant positive effect on the FCS of shrimp-producing households. In addition, it does not indicate a significant interaction effect. Therefore, it can be assumed that the positive effect of subsistence food consumption on the FCS applies to both: households that produce shrimps and households that do not produce shrimps. Likewise, ‘m2’ reveals a general significant positive effect of the share of market-bought (commercial) food consumption on the FCS. However, ‘m3’ reveals that the share of commercial food consumption has no significant effect on the FCS of shrimp-producing households. Interestingly, the effect is predicted to be significantly stronger, presumably even positive, for households that do not engage in shrimp production.

To shed light on the manifold varieties of livelihood strategies, we will discuss the results for these three variables together. Looking first at shrimp farmers in our sample, we note that their market dependency is slightly lower for those practicing crop diversification than those who do not. Together with the results from the regression model, this could support evidence that shrimp farmers consume relevant quantities of fish and other sources of protein from their own farms (Belton et al., 2014). However, while not part of the model, additional bivariate (Wilcoxon rank sum) tests provide indications that it is particular types of crop diversification (as in Appendix (Fig. 3)) which have a statistically significant effect on the FCS. Among shrimp farmers, those applying Rice & Golda (freshwater; FCS = 70.5**) and Rice & Fish (FCS = 70.4**) systems have significantly higher FCS in their household than farms that do not apply these combinations (FCS = 66.8 / FCS = 66.7).^4^ Taken together, these results could imply that it is those shrimp farmers also farming agricultural (staple) crops—enabled in part by less saline environments—that achieve the best FCS outcome. Second, our sample shows that among non-shrimp farmers, “other” crop diversification (e.g. mix of vegetables, pulses etc.), which is the most common crop diversification type here, has a negative effect (Wilcoxon: FCS = 57.9*** if applied, 60.4 if not). Furthermore, market dependency is notably higher and the share of food consumed from subsistence farming lower for those diversifying crops, than those that do not (Mann–Whitney U-Test: 0.000). This may suggest that the role of crop diversification among non-shrimp farmers may be geared towards diversifying the range of cash crops for the market rather than for own consumption (e.g. to spread risk of losing all income in case one crop fails). These results may be supportive of those by Sibhatu et al., (2015: 10,657) in Africa, who find that “on-farm production diversity is positively associated with dietary diversity in some situations, but not in all. When production diversity is already high, the association is not significant or even turns negative, because of foregone income benefits from specialization.” For coastal Bangladesh, Lázár et al. (2015) support that crop diversification into short duration vegetables, which are less affected by salinity than Boro rice, can often be more profitable (due to higher market prices) and hence a lucrative opportunity. Yet, they find rice-based systems less risky, i.e. more predictable due to their overall lower sensitivity to climate change effects. Non-shrimp farming households in our sample seem to have to purchase food items complementing and important to balanced diets, such as meat and fish, on the market. This is in line with Roos (2001), who found that fish purchased from local markets made up between 57–69% of consumed fish, depending on the season. Interestingly, this was true both for households with and without homestead fish ponds. That said, our data does indicate a possible positive effect of the mentioned aquaculture-agriculture combinations on FNS outcomes also for non-shrimp farmers, the majority of which reside in the freshwater zones: we found farms using Agroforestry (FCS = 68.7***) and Rice & Fish (FCS = 68.3**) to have significantly higher FCS in their household than farms that do not (FCS = 59.9 / FCS = 60).

Finally, ‘m2’ indicates a general significant positive effect of a wife being involved in the land use decision-making process on the FCS. Interestingly, ‘m3’ does not indicate a significant interaction effect. Therefore, it can be assumed that the positive effect of a wife being involved in the decision-making process applies in a more general manner, while in relative terms it is not of high importance, but significant. Women in Bangladeshi society are generally responsible for food procurement and preparation of meals for the household members (Ali, 2005). Hence, our findings support the literature (e.g. Sraboni et al., 2014) which posits that empowering women, including them in decision-making on household level and improving their access to resources such as land can positively contribute to household food security outcomes, thereby providing further and statistically tested evidence for the pathway identified by Kawarazuka and Bené (2010).

## Conclusions

The aim of this paper was to better understand the relative contribution of crop diversification to food and nutrition security outcomes—here, dietary diversity measured through the Food Consumption Score—among farming households in coastal Bangladesh. Our overall aggregated model does not indicate a significant contribution of crop diversification to improved dietary diversity. Rather, salinization and per capita household income show the strongest positive influence; weaker positive effects are observable through larger plot sizes, higher market dependency for food, higher shares of produced food for subsistence, and a wife being part of the decision-making process for land use.

Arguing that the relative importance and significance of factors contributing to the FCS are not overall generalizable, we provided a differentiated analysis between two distinct livelihood contexts in coastal Bangladesh: shrimp-based and agricultural crop-based farming systems, keeping in mind that crop diversification options in shrimp systems can be restricted due to the level of salinity in place. We found that households engaging in shrimp farming have a significantly higher dietary diversity than households that do not. Our results provide evidence for each of the three pathways between (fish-oriented) livelihoods and household FNS outcomes as identified in the literature by Kawarazuka and Bené (2010), i.e. own consumption, income generation and the positive effect of women’s empowerment on the household FCS (Harris-Fry et al., 2015; Sraboni et al., 2014).

The differentiated analysis of shrimp vs. non-shrimp farmers shows that crop diversification may well play a significant role in improving FNS. Among shrimp farmers, crop diversification shows the relatively strongest significant positive effect on dietary diversity. With market dependency for food among shrimp famers being lower for those applying crop diversification than those who do not, the analysis supports past evidence that shrimp farmers consume relevant quantities of fish and other sources of protein from their own farms, which are otherwise primarily market-oriented, i.e. selling shrimp for export, thereby generating lucrative incomes. By contrast, crop diversification seems to have a negative effect on dietary diversity among households that do not produce shrimp, especially when different agricultural crops are combined, possibly underscoring other studies showing that rice-based systems are less risky overall (Lázár et al., 2015). All in all, non-shrimp farmers have higher subsistence levels and lower market dependency for food, but seem to depend more on the market to significantly improve their dietary diversity. Non-shrimp farmers in the sample who diversify crops tend to increase the range of cash crops for the market while reducing produce for subsistence, thereby increasing market dependency.

That said, an important finding is that for both shrimp and non-shrimp farmers, it seems to be in particular those crop diversification systems combining agriculture with aquaculture, less saline systems such as in prawn (golda), and agroforestry which foster diverse diets among households. As such, our findings confirm that (direct) access to fish or aquaculture products as sources of protein and micronutrients is key to improving dietary diversity (Toufique & Belton, 2014). While by no means a panacea to solving FNS challenges among rural households and their agricultural problems caused by climate-change (McCord et al., 2015), we suggest that promoting crop diversification systems combining aquaculture and agriculture could be a beneficial pathway to improved FNS outcomes. At the same time, it is important to understand the context and rationales of the farmers in which this diversification takes place: FNS is affected by a multitude of factors, and income, for example, remains important.

Admittedly, a limitation of this analysis is that, like most other studies, we only claim correlations, not causalities, as we did not have a panel dataset. Further studies could address this through panel data or combine quantitative and qualitative approaches. Further, it will be interesting to study the effects of the discussed dynamics, including in particular, perhaps, the potentially changing role of off-farm activities to diversify livelihoods, in the context of Covid-19, which has hampered mobility (not only of labour migrants), but also access to markets for selling and buying food and produce.

## Data Availability

Data can be requested from the grant recipients.

## Appendix

**Table 3:** **Characteristics of unions** (Source: Bernzen et al., 2019)

District	*Union Parishad* (BBS code)	Polder number	2011 Pop	Pop. change 2001–2011 (%)	# HHs 2011	Distance to coast or next major river (km)	Bio-ecological zone	2011 Land Use (%) (NCA: Net Cultivated Area)	Land Zoning Classification	Natural hazards/ events
Khulna	Amadi 40/47/53/99/10	10–12	33,184	18.1	7434	0.3	Saline tidal floodplain	Agriculture = 61 Settlement = 16 Water body = 21 Golda/Bagda with white fish = 15	Agro-Aquaculture (Golda with White Fish) Zone	Soil salinity, cyclone-prone
Khulna	Bagali 40/47/53/99/11	13–14/1–2	33,027	13.9	8863	1.1	Saline tidal floodplain	Agriculture = 70 Settlement = 14 Water body = 26 Bagda-Golda with white fish = 32	Agro-Aquaculture (Bagda with white fish) Zone	Soil salinity, cyclone-prone
Bagerhat	Rampal 40/01/73/99/83	34/2	24,276	14.8	5840	3.5	Saline tidal floodplain	Agricul. = 25% of NCA River/canal = 11 Shrimp area = 75% of NCA Settlement = 14 Urban = 1	Urban and Commercial and Shrimp (Bagda) Zone	Soil salinity, cyclone-prone Aila: severe damage Sidr: moderate damage
Patuakhali	Lata Chapli 10/78/66/99/47	48	25,925	22.2	5835	1.1	Saline tidal floodplain	Agriculture = 54 Water = 05 Mangrove = 05 Sand = 02 Bay of Bengal = 16 Settlement = 18	Agro-Forestry (Mangrove) and Tourism Zone	Soil salinity, cyclone-prone Sidr: moderate-severe damage Mahashen: moderate-severe damage
Patuakhali	Itabaria 10/78/95/99/20	ITL	21,478	7.7	4490	0.5	Ganges floodplain	Agriculture = 50 River/canal = 17 Tidal flat = 03 Settlement = 30	Agro-Fisheries (open water, river, canal etc.) Zone	Riverbank erosion, cyclone-prone Sidr: severe damage Aila: moderate damage
Patuakhali	Deuli Subidkhali 10/78/76/99/27	47/1	32,169	15.0	5033	0.4	Ganges Floodplain	Agriculture = 48 Canal/river = 23 Settlement = 27 Urban = 2	Agro- Fisheries (open water, river, canal) Zone	Riverbank erosion, Cyclone-prone Sidr: severe damage Aila: moderate damage
Lakshmipur	Char Alexander 20/51/73/99/23	59/2, 59/2Ext	40,978	-23.3	8447	1.2	Meghna Estuarine Floodplain	Agriculture = 32 Settlement with homestead = 21 Water body = 43 Urban = 2 Tidal Flat = 2	Agro-Fisheries (Open water-river, khal etc.) Zone	Cyclone-prone, severe river bank erosion
Noakhali	Tamaruddin 20/75/36/99/95	73/1 A-B	27,979	10.9	6166	0.9	Offshore Islands	Agriculture- 43 Mangrove -07 settlement-02 Tidal flat—12 Water- 37	Agro-Forestry (Mangrove) Zone	Soil salinity, riverbank erosion, mildly affected by Sidr/Aila Severely affected by 1991 cyclone
Noakhali	Char Ishwar 20/75/36/99/28	73/1 A-B	26,228	-29.5	5381	1.5	Offshore Islands	Agriculture -54 Settlement—06 Tidal flat -10 Water -29 Mangrove—01	Agriculture Zone	Soil salinity, Riverbank erosion but also char land quickly rising, mildly affected by Sidr/Aila Severely affected by 1991 cyclone

**Table 4:** Average number and types of crops sold by shrimp farmers and non-shrimp farmers (multiple response possible)

	Shrimp-farmers	Non-shrimp-farmers
Average number of different crop types sold by household (min = 0, max = 4)	2.32	1.67
Crop type sold* Crops from aquaculture	Share (%) of HH selling crop	Share (%) of HH selling crop
Shrimp	96	0
Fish	89	7
Crab	0.006	0.004
Crops from agriculture / livestock		
Rice	26	52
Vegetables*	5	11
Fruit*	0	0.007
Pulses	2	57
Livestock and related products*	13	28
Oil seeds*	0	4
Other	0	0.01

Shrimp (*n* = 170), non-shrimp (*n* = 671). Source: BanD-AID survey data

*Open ended question, multiple response possible. Responses were regrouped as follows: Vegetables: vegetables, corn, potatoes, onions; Oil seeds: oil seeds, nuts, peanuts; Fruit: fruit, plums, watermelon; Livestock and related products: livestock and related products, chicken, buffalo, poultry, milk, eggs; Other: sugarcane, trees, seedlings, chili

**Table 5:** Descriptive Statistics of Independent Variables – Shrimp vs. Non-Shrimp Farming Households

Variable	mean shrimp	std. dev. shrimp	mean non-shrimp	std. dev. non-shrimp	significance of difference
General Demographics					
Age of HH (in years)	53.12	12.43	50.51	13.42	i) **
Education of HH (secondary and higher = 1)	0.30	0.46	0.16	0.37	ii) ***
Socio-economic Factors					
Primary income activity (off-farm = 1)	0.26	0.43	0.45	0.50	ii) ***
Household income per capita per month (in 100 taka)	46.97	33.88	30.28	27.02	i) ***
Physical Assets					
Accessible land (in sotansho)	806.83	1372.09	187.04	215.27	i) ***
Agricultural Strategies					
Crop diversification (yes = 1)	0.90	0.30	0.17	0.38	ii) ***
Land use changes between 2000 and 2015 (yes = 1)	0.60	0.49	0.16	0.37	ii) ***
Food Production and Consumption					
Share of subsistence food consumption (in %)	45.92	22.48	67.12	23.60	i) ***
Share of commercial food consumption (in %)	66.99	18.29	47.10	22.76	i) ***
Wife involved in land use decision making (yes = 1)	0.05	0.22	0.08	0.27	ii) ns
Environmental Risks					
Arable land lost (yes = 1)	0.02	0.14	0.24	0.43	ii) ***
Distance to closest river or coast (in km)	2.45	1.50	0.91	0.73	i) ***
Salinization (yes = 1)	0.66	0.48	0.16	0.37	ii) ***
Social Capital					
Time household has lived in village (in years)	48.47	16.63	43.04	17.72	i) ***
Neighbours most helpful in crisis (yes = 1)	0.44	0.50	0.22	0.42	ii) ***
NGOs most helpful in crisis (yes = 1)	0.16	0.37	0.54	0.50	ii) ***
Government most helpful in crisis (yes = 1)	0.44	0.50	0.68	0.47	ii) ***

Shrimp (*n* = 153), non-shrimp (*n* = 552); i) Mann–Whitney U-Test, ii) Chi2-Test

* significant at 10% level (*p* < 0.1); ** significant at 5% level (*p* < 0.05); *** significant at 1% level (*p* < 0.01). Source: BanD-AID survey data

## Footnotes

1 “Food and nutrition security exists when all people at all times have physical, social and economic access to food of sufficient quantity and quality in terms of variety, diversity, nutrient content and safety to meet their dietary needs and food preferences for an active and healthy life, coupled with a sanitary environment, adequate health, education and care” FAO/AGN (2011) *as cited* *in* Committee on World Food Security (2012)

2 When we refer to "shrimp zones" or "regions dominated by shrimp farming," we refer to the entire southwestern region of Bangladesh where we find a high concentration of extensive aquaculture operations, which include both (saltwater) shrimp (*bagda*) and/or (freshwater) prawn (*golda*) cultivation. Likewise, when we say “shrimp farmers” in the context of the analysis, these households may cultivate shrimp and/or prawn. We acknowledge that a further differentiation between these two groups can deliver even more detailed insights but are not within the scope of this paper.

3 Food groups and weight in FCS calculation: Main staples (2), pulses (3), vegetables (1), fruit (1), meat and fish (4), milk (4), sugar (0.5), oil (0.5), condiments (0). FCS calculation steps: (I) Using standard VAM (Vulnerability Analysis and Mapping) 7-day food frequency data, group all the food items into specific food groups. (II) Sum all the consumption frequencies of food items of the same group, and recode the value of each group above 7 as 7. (III) Multiply the value obtained for each food group by its weight which creates new weighted food group scores. (IV) Sum the weighed food group scores, thus creating the food consumption score (FCS). (WFP VAM 2008).

4 We cannot claim causal relationships for these results. All other observed crop diversification types in the sample were tested in the same manner but did not show statistically significant differences in the FCS.

## References

[CR1] Afroz T, Alam S (2013). Sustainable shrimp farming in Bangladesh: A quest for an integrated coastal zone management. Ocean and Coastal Management.

[CR2] Ahmed BN, Waibel H (2019). The role of homestead fish ponds for household nutrition security in Bangladesh. Food Security.

[CR3] Ahmed N (2013). Linking prawn and shrimp farming towards a green economy in Bangladesh: Confronting climate change. Ocean and Coastal Management.

[CR4] Akinwande M, Dikko H, Samson A (2015). Variance Inflation Factor: As a Condition for the Inclusion of Suppressor Variable(s) in Regression Analysis. Open Journal of Statistics.

[CR5] Ali, A. (2005). Livelihood and food security in rural Bangladesh. The role of social capital. PhD thesis, Wageningen University. https://library.wur.nl/WebQuery/wurpubs/fulltext/121729

[CR6] Anderman TL, Remans R, Wood SA, DeRosa K, DeFries RS (2014). Synergies and trade-offs between cash crop production and food security: A case study in rural Ghana. Food Security.

[CR7] Baliki G, Brück T, Schreinemachers P, Uddin MN (2019). Long-term behavioural impact of an integrated home garden intervention: Evidence from Bangladesh. Food Security.

[CR8] BBS, Bangladesh Bureau of Statistics (2011). Household income and expenditure survey 2010 Ministry of Planning.

[CR9] Belton B, Azad A (2012). The characteristics and status of pond aquaculture in Bangladesh. Aquaculture.

[CR10] Belton B, Bush SR (2014). Beyond net deficits: New priorities for an aquacultural geography. The Geographical Journal.

[CR11] Belton, B., Ahmed, N., Murshed-e-Jahan, K. (2014). Aquaculture, employment, poverty, food security and well-being in Bangladesh: A comparative study. Penang, Malaysia: CGIAR Program Report: AAS-2014–39.

[CR12] Belton B (2016). Shrimp, prawn and the political economy of social wellbeing in rural Bangladesh. Journal of Rural Studies.

[CR13] Béné C, Barange M, Subasinghe R (2015). Feeding 9 billion by 2050–Putting fish back on the menu. Food Security.

[CR14] Bernzen, A., Jenkins, J. C., & Braun, B. (2019). Climate change-induced migration in coastal Bangladesh? A critical assessment of migration drivers in rural households under economic and environmental stress. *Geosciences*, *9*(1), 51. 10.3390/geosciences9010051

[CR15] Berti PR, Krasevec J, Sian F (2004). A review of the effectiveness of agriculture interventions in improving nutrition outcomes. Public Health Nutrition.

[CR16] BFTI (2016). Bangladesh foreign trade institute, study on sector based need assessment of business promotion council- fisheries products.

[CR17] Carletto C, Zezza A, Banerjee R (2013). Towards better measurement of household food security: Harmonizing indicators and the role of household surveys. Global Food Security.

[CR18] Committee on World Food Security. (2012). Coming to terms with terminology: Food security, nutrition security, food security and nutrition, food and nutrition security. URL: http://www.fao.org/fsnforum/sites/default/files/file/Terminology/MD776(CFS___Coming_to_terms_with_Terminology).pdf

[CR19] Compact 2025. (2016). Bangladesh ending hunger & undernutrition challenges & opportunities. Scoping report for roundtable discussion. http://www.compact2025.org/files/2016/04/Bangladesh-Scoping-Report_Final.pdf (accessed 30 August 2020).

[CR20] Dey M, Spielman DJ, Haque AM, Rahman MS, Valmonte-Santos R (2013). Change and diversity in smallholder rice–fish systems: Recent evidence and policy lessons from Bangladesh. Food Policy.

[CR21] DFID. (2014). Can Agriculture Interventions Promote Nutrition? Agriculture and Nutrition Evidence Paper

[CR22] DoF, Department of Fisheries (2015). National fish week, compendium (In Bengali).

[CR23] DoF. Department of Fisheries. (2019). Yearbook of Fisheries Statistics of Bangladesh, 2018–19. Fisheries Resources Survey System (FRSS), Department of Fisheries, Bangladesh: Ministry of Fisheries and Livestock. 36: 135.

[CR24] E-Jahan KM, Ahmed M, Belton B (2010). The impacts of aquaculture development on food security: Lessons from Bangladesh. Aquaculture Research.

[CR25] FAO (2020). Second rapid assessment of food and nutrition security in the context of COVID-19 in Bangladesh: May – July 2020. Dhaka.

[CR26] FAOstat. (2020). Bangladesh. Hunger and Food Security. http://www.fao.org/faostat/en/#country/16 (accessed 30 August 2020)

[CR27] Fiorella KJ, Chen RL, Milner EM, Fernald LC (2016). Agricultural interventions for improved nutrition: A review of livelihood and environmental dimensions. Global Food Security.

[CR28] FSC, Food Security Cluster. (2009): Food Consumption Score (FCS) in Bangladesh Context, Technical Guideline January 2009. FAO and WFP. URL: http://fscluster.org/sites/default/files/documents/WFP_BAN_FCS%20technical%20guideline_Bangladesh%20context_Jan09.pdf

[CR29] FRSS. (2016). Fisheries statistical report of Bangladesh. Department of Fisheries, Bangladesh 32, 1–57.

[CR30] GED. (2015). Planning Commission Government of the People’s Republic of Bangladesh. Seventh five-year plan 2016 – 2020 Accelerating Growth, Empowering Citizens.

[CR31] Global Hunger Index. (2018). The Challenge of Hunger and Climate Change. Available at: https://www.globalhungerindex.org/ (Accessed 28 August 2020).

[CR32] Harris-Fry, H., Azad, K., Kuddus, A. et al. (2015). Socio-economic determinants of household food security and women’s dietary diversity in rural Bangladesh: a cross-sectional study. Available at: 10.1186/s41043-015-0022-0 (accessed 28 August 2020). 10.1186/s41043-015-0022-0 PMC5026026 26825273

[CR33] Heinze G, Wallisch C, Dunkler D (2018). Variable selection - A review and recommendations for the practicing statistician. Biometrical Journal.

[CR34] Herforth, A., Harris, J. (2014). Understanding and Applying Primary Pathways and Principles: Improving Nutrition through Agriculture. Technical Brief Series USAID/Strengthening Partnerships, Results, and Innovations in Nutrition (Spring).

[CR35] Hernandez R, Belton B, Reardon T, Hu C, Zhang X, Ahmed A (2018). The “quiet revolution” in the aquaculture value chain in Bangladesh. Aquaculture.

[CR36] Hufnagel J, Reckling M, Ewert F (2020). Diverse approaches to crop diversification in agricultural research. A Review. Agronomy for Sustainable Development.

[CR37] IFPRI. (2011). Leveraging Agriculture for Improving Nutrition and Health: Highlights from an International Conference. International Food Policy Research Institute (IFPRI), Washington, D.C.

[CR38] Islam AHMS, von Braun J, Thorne-Lyman AL, Ahmed AU (2018). Farm diversification and food and nutrition security in Bangladesh: Empirical evidence from nationally representative household panel data. Food Security.

[CR39] Jaenicke H, Virchow D (2013). Entry points into a nutrition-sensitive agriculture. Food Security.

[CR40] JPGSPH, James P Grant School of Public Health and National Nutrition Services. (2016). *State of food security and nutrition in Bangladesh 2015*. Dhaka, Bangladesh.

[CR41] Kadiyala S, Harris J, Headey D, Yosef S, Gillespie S (2014). Agriculture and nutrition in India: Mapping evidence to pathways. Annals of the New York Academy of Sciences.

[CR42] Karaca-Mandic P, Norton EC, Dowd B (2012). Interaction Terms in Nonlinear Models. Health Services Research.

[CR43] Kawarazuka N, Béné C (2010). Linking small-scale fisheries and aquaculture to household nutritional security: An overview. Food Security.

[CR44] Kremen C, Iles A, Bacon C (2012). Diversified farming systems: An agroecological, systems-based alternative to modern industrial agriculture. Ecology and Society.

[CR45] Kuma, T., Dereje, M., Hirvonen, K., Minten, B. (2018). Cash crops and food security: Evidence from Ethiopian smallholder coffee producers. *The Journal of Development Studies*, 1–18.

[CR46] Lázár, A.N., Clarke, D., Adams, H., Akanda, A.R., Szabo, S., Nicholls, R.J., Matthews, Z., Begum, D., Saleh, A.F., Abedin, M.A., Payo, A., Streatfield, P.K., Hutton, C., Mondal, M.S., Moslehuddin, A.Z. (2015). Agricultural livelihoods in coastal Bangladesh under climate and environmental change – a model framework. *Environmental Science: Process & Impacts*,* 17*, 1018–1031. 10.1039/c4em00600c 10.1039/c4em00600c 26034782

[CR47] Leroy JL, Ruel M, Verhofstadt E (2009). The impact of conditional cash transfer programmes on child nutrition: A review of evidence using a programme theory framework. Journal of Development Effectiveness.

[CR48] Lin B (2011). Resilience in Agriculture through Crop Diversification: Adaptive Management for Environmental Change. BioScience.

[CR49] Masset, E., Haddad, L., Cornelius, A., Isaza-Castro, J. (2012). Effectiveness of agricultural interventions that aim to improve nutritional status of children: systematic review. Bmj, 344, d8222. 10.1136/bmj.d8222 PMC3259800 22251864

[CR50] McCord PF, Cox M, Schmitt-Harsh M, Evans T (2015). Crop diversification as a smallholder livelihood strategy within semi-arid agricultural systems near Mount Kenya. Land Use Policy.

[CR51] Metcalfe I (2003). Environmental concerns for Bangladesh. South Asia: Journal of South Asian Studies.

[CR52] Murshed-E-Jahan K, Pemsl DE (2011). The impact of integrated aquaculture–agriculture on small-scale farm sustainability and farmers’ livelihoods: Experience from Bangladesh. Agricultural Systems.

[CR53] NNP, National Nutrition Policy. (2015). National Nutrition Policy, Nutrition is the Foundation to Development. Ministry of Health and Family Welfare. http://extwprlegs1.fao.org/docs/pdf/bgd152517.pdf (accessed 30 August 2020)

[CR54] Osmani S R (2016) Bangladesh: Issues in Contemporary Economics: Policy and Development 3, 170.

[CR55] Osmani, S. R., Ahmed, A. U,, Ahmed, T., Hossain, N., Huq, S., Shahan, A. (2016). Strategic review of food security and nutrition in Bangladesh. Dhaka, Bangladesh: World Food Programme (WFP). https://www.wfp.org/content/food-and-nutrition-security-bangladesh

[CR56] Oyarzun PJ, Borja RM, Sherwood S, Parra V (2013). Making sense of agrobiodiversity, diet, and intensification of smallholder family farming in the highland Andes of Ecuador. Ecology of Food and Nutrition.

[CR57] Pandey VL, Dev SM, Jayachandran U (2016). Impact of agricultural interventions on the nutritional status in South Asia: A review. Food Policy.

[CR58] Paul SK (2013). Post-cyclone livelihood status and strategies in coastal Bangladesh. Rajshahi University Journal of Life & Earth and Agricultural Sciences.

[CR59] Paul BG, Vogl CR (2011). Impacts of shrimp farming in Bangladesh: Challenges and alternatives. Ocean & Coastal Management.

[CR60] Paul BG, Vogl CR (2013). Organic shrimp aquaculture for sustainable household livelihoods in Bangladesh. Ocean & Coastal Management.

[CR61] Pierre-Louis JN, Sanjur D, Nesheim MC, Bowman DD, Mohammed HO (2007). Maternal income-generating activities, child care, and child nutrition in Mali. Food and Nutrition Bulletin.

[CR62] Pollet TV, van der Meij L (2017). To Remove or not to Remove: The Impact of Outlier Handling on Significance Testing in Testosterone Data. Adaptive Human Behavior and Physiology.

[CR63] Rahman MH, Lund T, Bryceson I (2011). Salinity impacts on agro-biodiversity in three coastal, rural villages of Bangladesh. Ocean & Coastal Management.

[CR64] Rahman MR, Ando K, Takeda S (2013). Effect of shrimp-based cropping systems on salinity and soil fertility in a coastal area of Bangladesh: A village-level study. Journal of Agricultural Science.

[CR65] Rampa, F., van Seters, J. (2013). Towards the development and implementation of CAADP regional compacts and investment plans: The state of play. European Center for Development Policy Management (ECDPM), Maastricht, The Netherlands and Brussels, Belgium.

[CR66] Rashid S, Minot N, Lemma S (2016). Does a “blue revolution” help the poor? Evidence from Bangladesh. Agricultural Economics.

[CR67] Reardon T, Chen KZ, Minten B (2014). The quiet revolution in Asia's rice value chains. Annals of the New York Academy of Sciences.

[CR68] Ruel MT, Alderman H (2013). Nutrition-sensitive interventions and programmes: How can they help to accelerate progress in improving maternal and child nutrition?. The Lancet.

[CR69] Roos, N. (2001). Fish consumption and aquaculture in rural Bangladesh: Nutritional contribution and production potential of culturing small indigenous fish species (SIS) in pond polyculture with commonly cultured carps. Ph.D. Thesis, Department of Human Nutrition, The Royal Veterinary and Agricultural University, Denmark

[CR70] Roy A, Basu S (2020). Determinants of Livelihood Diversification Under Environmental Change in Coastal Community of Bangladesh. Asia-Pacific Journal of Rural Development..

[CR71] Shamsuzzaman MM, Islam MM, Tania NJ, Al-Mamun,  (2017). Fisheries resources of Bangladesh: Present status and future direction. Aquaculture and Fisheries.

[CR72] Sibhatu KT, Krishna VV, Qaim M (2015). Production diversity and dietary diversity in smallholder farm households. Proceedings of the National Academy of Sciences.

[CR73] Sohel MSI, Ullah MH (2012). Ecohydrology: A framework for overcoming the environmental impacts of shrimp aquaculture on the coastal zone of Bangladesh. Ocean & Coastal Management.

[CR74] Sohns F, Revilla Diez J (2017). Self-Employment and Its Influence on the Vulnerability to Poverty of Households in Rural Vietnam—A Panel Data Analysis. Geographical Review.

[CR75] Sraboni E, Malapit HJ, Quisumbing AR, Ahmed AU (2014). Women’s Empowerment in Agriculture: What Role for Food Security in Bangladesh?. World Development.

[CR76] Steyn N, Nel J, Nantel G, Kennedy G, Labadarios D (2006). Food Variety and Dietary Diversity Scores in Children: Are they Good Indicators of Dietary Adequacy?. Public Health Nutrition.

[CR77] Swapan MSH, Gavin M (2011). A desert in the delta: Participatory assessment of changing livelihoods induced by commercial shrimp farming in Southwest Bangladesh. Ocean and Coastal Management.

[CR78] Thorne-Lyman AL, Valpiani N, Sun K (2010). Household Dietary Diversity and Food Expenditures Are Closely Linked in Rural Bangladesh, Increasing the Risk of Malnutrition Due to the Financial Crisis1–3. The Journal of Nutrition.

[CR79] Toufique KA, Belton B (2014). Is aquaculture pro-poor? Empirical evidence of impacts on fish consumption in Bangladesh. World Development.

[CR80] Toufique KA, Gregory R (2008). Common waters and private lands: Distributional impacts of floodplain aquaculture in Bangladesh. Food Policy.

[CR81] Webb-Girard A, Cherobon A, Mbugua S, Kamau-Mbuthia E, Amin A, Sellen DW (2012). Food insecurity is associated with attitudes towards exclusive breastfeeding among women in urban Kenya. Maternal and Child Nutrition.

[CR82] Webb P, Kennedy E (2014). Impacts of agriculture on nutrition: Nature of the evidence and research gaps. Food and Nutrition Bulletin.

[CR83] WFP VAM, World Food Programme Vulnerability Analysis and Mapping Branch. (2008). Food Consumption Analysis. Calculation and Use of the Food Consumption Score in Food Security Analysis. Rome.

[CR84] Wiesmann, D., Bassett, L., Benson, T., Hoddinott, J. (2009). Validation of the World Food Programme’s Food Consumption Score and Alternative Indicators of Household Food Security. Discussion Paper. IFPRI.

[CR85] World Bank. (2018). Bangladesh: Reducing Poverty and Sharing Prosperity. https://www.worldbank.org/en/results/2018/11/15/bangladesh-reducing-poverty-and-sharing-prosperity (accessed 30 August 2020)

[CR86] WorldFish. (2017). WorldFish in Bangladesh. Factsheet: 2017–14. Penang, Malaysia: WorldFish.

